# Th17-Related Cytokines as Potential Discriminatory Markers between Neuromyelitis Optica (Devic’s Disease) and Multiple Sclerosis—A Review

**DOI:** 10.3390/ijms22168946

**Published:** 2021-08-20

**Authors:** Karina Maciak, Sylwia Pietrasik, Angela Dziedzic, Justyna Redlicka, Joanna Saluk-Bijak, Michał Bijak, Tomasz Włodarczyk, Elzbieta Miller

**Affiliations:** 1 Department of General Biochemistry, Faculty of Biology and Environmental Protection, University of Lodz, 90-236 Lodz, Poland; karina.maciak@edu.uni.lodz.pl (K.M.); sylwia.pietrasik@edu.uni.lodz.pl (S.P.); angela.dziedzic@edu.uni.lodz.pl (A.D.); joanna.saluk@biol.uni.lodz.pl (J.S.-B.); 2 Department of Neurological Rehabilitation, Medical University of Lodz, 93-113 Lodz, Poland; justyna.redlicka@umed.lodz.pl; 3 Biohazard Prevention Centre, Faculty of Biology and Environmental Protection, University of Lodz, 90-236 Lodz, Poland; michal.bijak@biol.uni.lodz.pl; 4 Department of Ophthalmology, Third Municipal Hospital Dr. Charles Jonscher Hospital of Lodz, 93-113 Lodz, Poland; tomaszwlodarczyk78@gmail.com

**Keywords:** neuromyelitis optica, Devic’s disease, multiple sclerosis, biomarkers, Th17-related cytokines

## Abstract

Multiple sclerosis (MS) and Devic’s disease (NMO; neuromyelitis optica) are autoimmune, inflammatory diseases of the central nervous system (CNS), the etiology of which remains unclear. It is a serious limitation in the treatment of these diseases. The resemblance of the clinical pictures of these two conditions generates a partial possibility of introducing similar treatment, but on the other hand, a high risk of misdiagnosis. Therefore, a better understanding and comparative characterization of the immunopathogenic mechanisms of each of these diseases are essential to improve their discriminatory diagnosis and more effective treatment. In this review, special attention is given to Th17 cells and Th17-related cytokines in the context of their potential usefulness as discriminatory markers for MS and NMO. The discussed results emphasize the role of Th17 immune response in both MS and NMO pathogenesis, which, however, cannot be considered without taking into account the broader perspective of immune response mechanisms.

## 1. Introduction

Multiple sclerosis (MS) is a chronic inflammatory and neurodegenerative disease, characterized by the infiltration of immune cells into the central nervous system (CNS) with subsequent demyelination, axonal damage, and neuronal loss. The disease shows great heterogeneity concerning radiological and histopathological changes, as well as clinical appearance and progression. Recently, many disease entities have been identified to originate from MS and have been often confused with it. One of them is neuromyelitis optica (NMO; Devic’s disease), an immune-mediated chronic inflammatory disease of the CNS with clinical and radiological features overlapping with MS. Although it was considered a clinical variant of MS for years, it is now regarded as a distinct disease entity. According to the newest version of the International Statistical Classification of Diseases and Related Health Problems, adopted by the World Health Organization (WHO), MS and NMO belong to a large group of white matter diseases. However, they are classified as separate, with different radiological features [1]. NMO is an autoimmune disorder, mediated in most cases by antibodies against the aquaporin (AQP)-4 water channel [2]. For the majority of patients, antibodies against AQP4 can be detected with an immunoassay [3]. The latest criterion of diagnostic guidelines unifies AQP4-negative and positive forms under the umbrella of neuromyelitis optica spectrum disorders (NMOSDs) [4].

The advent of NMO antibodies has permitted clearer differentiation between NMO and MS. Constructing a definitive and correct diagnosis of NMO can be challenging, because a prevailing early stage of MS (relapsing-remitting phase) may manifest similar clinical symptoms (e.g., with attacks of optic neuritis and myelitis) [5]. It is essential to distinguish these two conditions, since patients with NMO require long-term immunosuppression to prevent devastating relapses. Disease-modifying treatments for MS such as interferon (IFN)-β may be inefficient in NMO or even worsen the disease course [6,7]. Hence, there is a great need to identify good markers to help promptly differentiate MS from NMO.

One way to discriminate them is based on immunological profiles, since these two disorders prove different immunopathogenesis. Both MS and NMO are autoimmune diseases characterized by chronic inflammation of the CNS and mostly relapsing course [8,9,10,11]. The pathological inflammation in autoimmune diseases results from complicated interactions between all cells of the adaptive and innate immune systems. The main contribution to this process is attributed to CD4+ T cells, specifically to the dysregulation between effector Th and Treg cells activity and the development of autoreactive effector Th cells [12]. The pathogenesis of MS was initially thought to be strictly related to the imbalance between a pro-inflammatory subset of Th1 cells and anti-inflammatory Th2 cells, which are responsible for recovery from the disease. Th1 cells may contribute in two different ways to the MS pathogenesis, as IFN-γ-producing cells that activate macrophages in the degradation of the myelin sheath, as well as a cells that directly impair the myelin sheath through the TNF-α release. Whereas, Th2 cells by an upregulation of anti-inflammatory cytokines, such as IL-4, IL-10 and TGF-β play a crucial role in inhibition of MS course [13]. However, subsequent studies have re-evaluated this view, suggesting that it is too highly polarized as it depends on only one effector population [14]. Despite the undeniable role of the Th1/Th2 interplay, more recently, Th17 cells appeared at the center of interest in the context of the pathogenesis of many inflammatory autoimmune diseases [15,16]. Interestingly, research results demonstrated the relevance of Th17 not only in MS but also in NMOSDs immunopathogenesis [17,18]. Understanding distinct molecular mechanisms of both conditions may be crucial to identifying specific discriminatory biomarkers for them. This review aims to discuss crucial differences in immune response associated with Th17 in the context of the overall immunopathogenesis of MS and NMOSD.

## 2. Similarities and Differences between MS and NMO

### 2.1. Epidemiology

Both MS and NMO have various prevalence primarily dependent on the geographic location, gender, and age. Globally, the average value for MS is estimated at 35.9 per 100,000 people [19], whereas the prevalence of NMO ranges from 0.52 to 4.4 per 1,000,000 people [20]. NMO is a notably rare disease compared to MS. Both diseases have been identified worldwide, however, in the European and North American populations the prevalence of MS is over 100 times higher than in the case of NMO (1 per 100,000 people) [21,22]. In NMO, the female-to-male ratio remarkably exceeds 3:1 (ratio reported in MS) and reaches 9:1 in some seropositive patients [23,24]. MS, as well as NMO, can occur at any age but appears mainly among young adults. The median age of the onset of NMO is 39 years, while for MS patients, the first manifestation of the disease occurs earlier—the mean age at the onset is 29 years [25,26,27].

### 2.2. Clinical Manifestations

The clinical courses of MS were first defined in 1996 [28] and updated in 2013 [29] to provide an assessment of the current disease status. From 2013, four clinical phenotypes are used to characterize MS: clinically isolated syndrome (CIS, single phase episode of CNS demyelination in a patient who has never been diagnosed with MS), relapsing-remitting MS (RRMS), primary-progressive MS (PPMS), and secondary-progressive MS (SPMS) [30]. There is a worse prognosis once MS patients enter a progressive phase, whether from the onset (like in PPMS) or after an initial RR phase. Most RRMS patients have only a moderate disability (Expanded Disability Status Scale (EDSS) ≤ 4.0), while in SPMS patients, severe and persistent disability develops (EDSS ≥ 5.5) [31]. The longer the disease duration, the greater the proportion of patients converting to a secondary-progressive phase, with a near 90% chance of conversion after 26 years from the onset [32]. Despite many factors predisposing to the transition to the progressive phase, such as older age at the onset, more frequent and severe attacks, patients with NMO compared to MS still have a low conversion rate to secondary progressive type [33].

In contrast to MS, NMO is an idiopathic, severe, demyelinating disorder of the CNS with a tendency to affect the optic nerve and longitudinally extensive transverse myelitis (LETM) with relative bypassing the brain injury [34]. In NMO, more than 90% of cases have a relapsing course with optic nerve attack, myelitis or both, occurring unpredictably [35]. About 10% of NMO patients have a monophasic course and they are more often associated with simultaneous optic myelitis, while a progressive course is extremely rare [36]. Untreated attacks of optic myelitis are often more disabling than in MS, leading to a faster accrual of irreversible neurological disability. After 7–8 years from the onset of disease, approximately 60% of NMO patients show severe motor impairment (EDSS ≥ 6) and at least blindness in one eye [36]. The vast majority of NMO-related deaths result from acute ascending myelitis or brainstem involvement causing respiratory failure [37]. NMO is a typical relapsing disease with multiple attacks leading to accumulating neurological injury and irreversible disability [38]. The brain magnetic resonance imaging (MRI) in NMO may be normal or show relatively mild changes, whereas the optic nerve may present areas of abnormality. NMO commonly affects only the optic nerve and the spinal cord, while MS mainly attacks the brain, as well as the spinal cord but less often than the optic nerve. In MS, the spinal cord lesions are usually a short segment (one vertebra long or shorter) located peripherally [39], while in NMO, the MRI scan shows longitudinally extensive (more than three) vertebral segment lesions with prominent involvement of the central cord [40].

### 2.3. Pathological Manifestations

A cellular autoreactive immune response involving a repertoire of CD4+ Th cells, particularly Th1 and Th17, is considered characteristic of MS onset and progression, whereas regulatory cytokines such as interleukin (IL)-10 and transforming growth factor (TGF)-β and Th2 cells, releasing IL-4, IL-5, IL-13 and IL-25, are related to anti-inflammatory effects and recovery [41]. Cytokines promote the differentiation of Th1 and Th17 cells, which subsequently infiltrate into the CNS and act via glial cells to induce the production of inflammatory agents, chemokines, matrix metalloproteinases, and free radicals [42]. Th1 cells produce mainly pro-inflammatory cytokines, such as IL-2, tumor necrosis factor (TNF)-α, and signature interferon (IFN)-γ, which activates the resident macrophages of the CNS, microglia, and stimulate their polarization towards pro-inflammatory M1-like phenotype [43]. Immunopathogenesis driven by Th1 cells is strictly associated with Th17 cells, which are susceptible to the inhibition by IFN-γ, recruit other inflammatory cells (mainly neutrophils) to the target tissue [44,45], and are the main producers of proinflammatory cytokines IL-17A and IL-17F [46]. Apart from suppressing Th17, IFN-γ inhibits CXCL2 [47] and matrix metalloproteinase (MMP)-9 [48], recognized to be induced by IL-17A and IL-17F [49,50]. Secondary inflammatory infiltrates in MS are dominated by IFN-γ-activated macrophages, whereas neutrophils, attracted by IL-17A–mediated chemokines, are scarce in MS lesions and absent in the CSF [51].

Th1/Th17 interplay, remaining under the control of particular cytokines, is crucial in driving tissue-specific autoimmunity [12]. Cascades of reactions lead to the modulation of glial cells and astrocytes activity, and neuroinflammation resulting in demyelination, axonal loss, and neurodegeneration [52,53,54]. Interestingly, it has been suggested that the profiles of Th1 and Th17-related immune response may evolve across the progression of MS [42,55]. Th17 cells might be stronger inducers of demyelination than Th1, however, there is a need for further studies to determine whether the Th17-driven EAE exhibits a different course than the Th1-induced one. Few studies conducted in MS demonstrated that the relapsing-remitting phenotype of the disease with prevailing spinal cord lesions was mainly driven by Th17 cells. In turn, MS phenotype with predominant brain lesions was recognized as associated with Th1 cells [56]. Research conducted on the EAE model suggested that the brain may be more susceptible to inhibitory signaling of IFN-γ than the spinal cord, which means Th17 cells/IL-17 might overcome the inhibitory effect of IFN-γ [56]. Moreover, it has been revealed that the determinant of this effect was a Th17/Th1 ratio—the high amount of Th17 did not trigger brain inflammation until the amount of Th1 was higher in the infiltrating population [56]. Together, it seems that these findings may provide new directions for further studies on distinguishing MS from MS-originated disease entities, such as NMO/NMOSD and predicting their clinical presentation.

**Figure 1:**
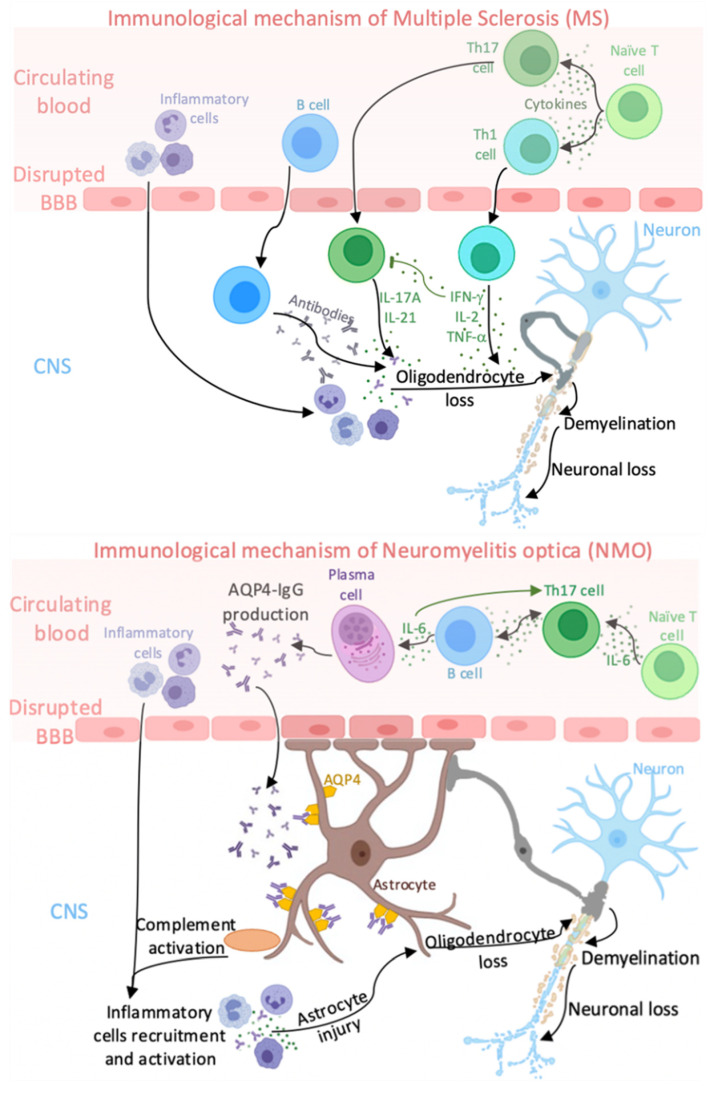
Immunological mechanism of MS and NMO. In MS: T helper (Th)-17 and Th1 cells differentiate from naïve T cells. The differentiation is stimulated by interleukin (IL)-1, IL-6, IL-12, IL-18, and IL-23. Th17 and Th1 cells alongside B cells infiltrate the central nervous system (CNS) at areas of increased blood–brain barrier (BBB) permeability. Herein, Th1 and Th17-related cells produce mainly pro-inflammatory cytokines which induce inflammatory cells infiltration. Immunopathogenesis driven by Th1 cells is strictly associated with Th17 cells, which are susceptible to the inhibition by IFN-γ. Moreover, B cells produce antibodies reactive against myelin antigens. Subsequently, inflammatory cells alongside cytokines and antibodies lead to oligodendrocyte loss, demyelination, and finally, neuronal loss. In NMO: Th17 cells differentiate from naïve T cells. The differentiation is stimulated by transforming growth factor (TGF)-β, IL-1β, IL-6, IL-7, IL-21, and IL-23. Th17 cells, inducing pro-inflammatory cytokines, including IL-6, interact with B cells, which stimulate plasma cells to produce aquaporin (AQP4)-4-IgGs. Besides, increased level of IL-6 associates with the tendency to release more IL-17A by Th17 cells and the proper differentiation of Th17 cells depends on IL-6 and IL-21 high level. AQP4-IgGs infiltrate into the CNS at areas of increased BBB permeability and then bind selectively to AQP4 on astrocytic endfeet. This binding induces the complement activation, which leads to the recruitment of peripheral inflammatory cells. Subsequently, inflammatory cell recruitment and activation alongside cytokines cause astrocyte injury, oligodendrocyte loss, demyelination, and finally, neuronal loss.

NMO is an astrocytopathy with the distinctive presence of AQP4 autoantibodies and complement activation with loss of immunoreactivities for the astrocytic proteins, AQP4 and glial fibrillary acidic protein (GFAP) [57]. The fact that 10–25% of NMO patients are AQP4-IgG-seronegative provides that the immunopathogenesis of the disease is heterogeneous and may involve other factors, such as antibodies against myelin oligodendrocyte glycoprotein (MOG-IgGs) and autoantibodies against aquaporin-1 (AQP1-IgGs) [58]. In contrast to MS, a B cell-mediated humoral autoimmunity supported by Th2 is thought to predominate in NMO patients [59]. However, research results suggest that the delineation of immune response in both diseases is not clear. The main differences between the immune response in MS and NMO are illustrated in Figure 1.

At the NMO onset, AQP4-IgGs infiltrate the CNS through the disrupted BBB or via endothelial transcytosis and bind to a protein AQP4. It leads to down-regulation of the surface AQP4 and disruption of water homeostasis in the CNS, activation of the astrocytes-derived complement, increased permeability of BBB, and infiltration of neutrophils and eosinophils into the CNS. This cascade results in demyelination, damage of oligodendrocytes, astrocytes, and neurons [58].

The inflammatory cells associated with NMO are primarily granulocytes (eosinophils and neutrophils) and macrophages, with a small number of lymphocytes observed, whereas the inflammatory process of MS is rich in lymphocytes and macrophages [60,61]. In the CSF of NMO patients, granulocytes and a high level of granulocyte colony-stimulating factor (G-CSF) were identified [61,62]. It has been demonstrated on the animal model that neutrophils are responsible for enhancing the severity of NMO lesions [63]. Macrophages produce pro-inflammatory cytokines and contribute to myelin phagocytosis, which leads to axonal damage [64]. Other relevant factors involved in the innate immune response in NMO are natural killer (NK) cells inducing antibody-dependent cellular cytotoxicity (ADCC) [62]. Plasmablasts are the primary source of AQP4-IgG, and they are affected by IL-6, which promotes the production of this autoantibody during NMO [65]. According to research results, T cells contribute to the NMO development only indirectly, e.g., by inducing cytokines that mediate antibody production and recruitment of granulocytes into the CNS [61]. As mentioned before, the relevant involvement in the NMO pathogenesis is also attributed to Th17 cells. This aspect is discussed in detail below.

### 2.4. Environmental and Genetic Factors

It is believed that factors contributing to the variation in the prevalence, either on NMO and MS, could be the environmental and genetic agents [66]. The most reported risk factors involved in the MS etiology are smoking, exposure to infectious diseases (e.g., Epstein–Barr virus), UV exposure, vitamin D deficiency, lifestyle, diet, and hormonal issues [67]. Similar environmental factors are associated with increasing the risk of developing NMO, among which are smoking, alcohol consumption, physical inactivity, low intake of dairy products, fish, vitamin D, and iron [68]. Many genetic factors contributing to MS have been identified in the European population. The primary susceptibility allele is a human leukocyte antigen (HLA) class II gene. The main one is HLA-DRB1*15:01, a well-known risk factor for MS carried by 25–30% of the population in northern Europe and the USA, which shows the strongest association with MS amongst all classical and single nucleotide polymorphism (SNP) alleles [69]. Several studies reported associations of some HLA alleles with NMO susceptibility in various races. In East Asian populations, HLA-DRB1*16:02 (OR = 8.39, *p* < 0.001), HLA-DRB1*08:02 (OR = 2.86, *p* < 0.0001), HLA-DPB1*05:01 (*p* = 0.0368) and HLA-DRB1*09:01 (OR = 0.27, *p* < 0.0001) are thought to be risk factors and a protective factor, respectively [70,71].

### 2.5. Treatment

Generally, the treatment regime in MS and NMO can be divided into two strategies: modifying the disease progression and treating the acute relapse [72]. The first line of treatment is the same for MS and NMO patients. Administration of high-dose intravenous methylprednisolone (up to 1000 mg daily) over a period of 3 to 5 days commonly represents the first step in the acute relapse treatment to reduce the inflammation. Additionally, glucocorticoids administration may downregulate cellular cytotoxicity and lead to the death of activated B cells [73]. However, recent studies have underlined the issue of misdiagnosis of MS with NMO and its possible harmful consequences [74,75]. Particular attention should be paid to the fact that several approved disease-modifying therapies (DMTs) that are applied in MS treatment have been found ineffective and even harmful to NMO patients [76], including IFN-β [77], natalizumab [78], fingolimod [79], and alemtuzumab [80]. This emphasizes the need for an accurate distinction between these two disorders [81].

Most recently, Agasing et al. described the mechanism by which cascade involving IFN type I (including IFN-β and the various IFN-α molecules), IL-6, and B-cells drive Th1 and Th17-mediated autoimmunity in NMOSD. They suggested that IFN-I stimulates the production of IL-6 by B cells, subsequently driving the pathogenic Th17 differentiation in vitro [82]. This fact demonstrates the significant involvement of Th1 and Th17 in the pathogenesis of NMO. Therefore, before introducing treatment for MS, it is crucial to the first rule out NMO by performing serological tests for anti-AQP4 and anti-myelin oligodendrocyte glycoprotein (MOG) presence in all patients with features characteristic for NMO [83]. Therefore, an early implementation of proper treatment is essential for its efficacy. That is why it is so important to the exact disease diagnosis. The most used immunosuppressants in NMO include: the oral drugs azathioprine (reducing relapse rates and improving visual scores [84]), mycophenolate mofetil (inhibition of inosine monophosphate dehydrogenase [85]), and rituximab (an intravenous anti-CD20 monoclonal antibody [86]).

The differences between MS and NMO are summarized in Table 1.

## 3. Brief Immunology of MS and NMO

IL-17A is a potent pro-inflammatory cytokine produced by both Th17 cells and IL-17-secreting CD8+ T cells. It contributes to the pathophysiology of autoimmune disorders and possibly mediates delayed-type inflammation by inducing chemokine-related recruitment of neutrophils and monocytes to the inflammatory location. The role of this cytokine in EAE is well-documented [91]. IL-17A and IL-17F may be produced as an effect of the phagocytosis of neutrophils by microglia and macrophages and further IL-23 release. In addition, IL-17A and IL-17F induce a lot of immune cells to release G-CSF, which results in the production of neutrophils and chemotaxis, and subsequently the elevation of activated neutrophils product, myeloperoxidase (MPO) [92,93,94].

IL-6 signaling promotes the pro-inflammatory activity of T-cells by inducing Th17 differentiation and production of pathogenic AQP4-IgG. In consequence, it contributes to disruption of the BBB and infiltration of inflammatory cells into the CNS [95]. Neuronal cells, microglia, astrocytes, and endothelial cells release this cytokine in response to injury. IL-6 is increased under neuroinflammatory conditions, which leads to demyelination and damage of axons and oligodendrocytes [96]. It has been shown that IL-6 levels correlate with AQP4-IgG and glial fibrillary acidic protein concentration, which is found to be a sign of astrocytic damage [97]. Moreover, this cytokine is considered a regulator of the balance between Th17 and regulatory T cells (Treg) [98].

### 3.1. Significance of Th17 in MS and NMO

The identification of effector Th17 and regulatory T (Treg) cells in the context of autoimmunological diseases allowed for the expansion of knowledge and to resolve some inadequacies of the classical Th1/Th2 concept that had dominated in T cell immunology for over 20 years [99]. A few studies have implicated CD4+ Th17 cells and their cytokines and downstream pathways in the pathogenesis of CNS autoimmunity in MS [42,55] and NMO [100,101], challenging the classical Th1/Th2 paradigm. In contrast to the conventional Th1 and Th2 cells, which represent stably polarized subsets, Th17 cells display an extraordinary heterogeneity (in terms of trafficking receptors), instability (when they cease to express their signature cytokine IL-17A), and plasticity (when they start expressing cytokines typical of other lineages) upon in vitro re-stimulation [102,103]. Memory Th17 cells play a significant role in the occurrence of relapses and the progression of MS [104] and NMO [105].

Th17 phenotype, differentiated from naïve CD4+ T cells, was first reported by the production of IL-17A (a member of the IL-17 family including IL-17A-F) [106]. The development, differentiation, and maturation of Th17 are stimulated by TGF-β, IL-1β, IL-6, IL-7, IL-21, and IL-23, which are produced by antigen-presenting cells (APCs) [16]. The Janus kinase (JAK)/activator of transcription 3 (STAT3) pathway is stimulated by cytokines, which results in the upregulation of transcription factors retinoic acid receptor-related orphan receptor α and γt (RORα and RORγt), critical for Th17 differentiation [107]. Th17 induces pro-inflammatory cytokines, such as IL-6, IL-8, IL-9, IL-21, IL-22, IL-23, IL-26, TNF-α, granulocyte macrophage colony-stimulating factor (GM-CSF), and G-CSF [24]. The differentiation of Th17 cells is inhibited mainly by cytokines released by Th1 and Th2. Among them, IL-2 is considered the critical inhibitor due to its important role in the activation of transcription factor signal transducer and activator of transcription 5 (STAT5) [108].

Although MS was initially considered the disease primarily associated with Th1, studies confirmed an essential commitment of Th17 in its pathophysiology. The level of Th17 cells in the CSF was elevated during MS exacerbation, unlike Th1 cells [109]. The subsequent studies demonstrated increased Th17 cells and IL-17A in the CSF, brain tissue, and peripheral blood in RRMS, especially during the relapse phase, compared to non-inflammatory neurological diseases and controls [109]. GM-CSF, a growth factor, which acts as a pro-inflammatory cytokine involved in Th17 functioning, was increased in the CSF and blood in MS patients but not in NMO [97]. In patients with NMO, Th17-related cytokines were found in active lesions and reported to be increased in serum and CSF, suggesting their involvement in the NMO pathogenesis [97,110]. Th17 cells and Th17-related cytokines interact with B cells producing antibodies and induce lesions in CNS [110]. It has also been revealed that AQP4-specific T cells support the Th17 operation in the pathomechanism of NMO [111]. Production of Th17-related cytokines IL-6 and IL-21 was increased in NMO patients in the remission phase. It was directly linked with a neurological disability as well as more robust resistance to immunomodulatory treatment with glucocorticoid [112]. NMO-IgG enhanced a positive-feedback loop of IL-6 expression involving signaling pathways such as JAK/STAT and MAPK. The production and gene expression of IL-6 in astrocytes were increased after stimulation by NMO-IgG, and inhibition of the IL-6/JAK/STAT3 pathway by specific inhibitor JAK1/2 reduced this effect [113]. It is possible that B cells, serving as APCs in the context of NMO and being a source of IL-6, are capable of promoting T cell polarization towards Th17 [114].

### 3.2. Differences in Th17-Related Markers between MS and NMO

Wang et al. revealed that both in MS and NMO patients, Th1-, Th2-, and Th17-related cytokines participated in the inflammation process. Pro-inflammatory IL-2 and IFN-γ were found significantly elevated in the serum of NMO patients compared to MS, as well as anti-inflammatory IL-4 and IL-10. Inflammatory changes induced by TNF-α and IL-17A were similar in NMO and MS patients [9]. In the same study, the optimal cutoff point of IL-2 ≥ 5 pg/mL has been suggested as a potentially useful biomarker for distinguishing NMO from MS [9]. Markers that were increased among NMO/NMOSD patients include many factors, such as IL-6, IL-17A, G-CSF, High Mobility Group Box 1 Protein (HMGB1), IL-21, IL-8, IL-13, GFAP and osteopontin (OPN) [115].

A recently conducted meta-analysis included 38 clinical trials has shown that the proportion of Th17 cells was much higher in NMOSD patients than in control (*p* = 0.013) and MS (*p* = 0.023) groups, as well as revealing the existence of different profiles of Th17-derived pro-inflammatory cytokines between NMO and MS [17]. In one study, Hou et al. demonstrated that the levels of IL-6 in CSF and serum and IL-17A in plasma and serum were higher in NMOSD patients than in MS patients [17]. Increased levels of IL-17A and IL-17F were considered both in the CSF [92] and in the blood [18] of NMO patients. Matsushita et al. focused on comparing NMO, RRMS, and PPMS and suggested that the elevated levels of Th17-related cytokines are characteristic of NMO/NMOSD [116]. They also reported significant differences in the level of pro-inflammatory cytokines, particularly IL-6, IL-17A, and G-CSF in CSF between NMO, RRMS, and PPMS. In NMO/NMOSD patients, IL-17A and IL-6 levels in CSF were meaningfully higher than in patients with RRMS (*p* = 0.024 and *p* = 0.012, respectively). In NMO/NMOSD, both levels of IL-6 and IL-17A were increased compared to RRMS patients (IL-6: *p* = 0.012; IL-17A: *p* = 0.024). As well as CSF, concentration of IL-6 was higher in NMO/NMOSD patients than in PPMS patients (*p* = 0.020). Moreover, in RRMS patients, IL-6 levels in CSF were significantly higher than in non-inflammatory neurological diseases (OND) patients being at the relapse phase. Additionally, a positive correlation between the levels of IL-6 and G-CSF and maximal spinal cord lesion length in the relapse phase in NMO/NMOSD patients is observed (IL-6: r = 0.47, *p* = 0.035; G-CSF: r = 0.47, *p* = 0.038), while in the remission phase, no significant correlation between clinical parameters and cytokine levels in RRMS or NMO/NMOSD was reported [116]. It has been demonstrated that the level of Th17 cells, IL-17A-secreting CD8+ T cells, and serum IL-17A levels are increased in NMO patients in the acute phase of the disease, compared to MS patients and healthy controls. Wang et al. have demonstrated that the mean concentration of IL-17A in patients with NMO was much higher than in patients with MS (*p* = 0.027) [105]. It could be associated with the increased severity of the neuroinflammation and demyelinating processes in NMO patients. The activity of IL-17A contributes to infiltration of the inflammatory cells through the spinal vessels, which causes lesions not observed in typical MS. The concentration of IL-17A and G-CSF (in addition to MPO, CCL11, and CCL4) in post-acute serum allowed to distinguish NMO from MS patients, which may be associated with the activity and differentiation of neutrophils and eosinophils chemotaxis [117].

IL-21 is primarily produced by CD4+ T cells, such as Th17 and follicular helper T cells, and NK cells [118,119]. On the other hand, Wurster et al. have shown that IL-21 is preferentially produced by derived Th2 effector cells that modulate the development of IFN-γ-producing Th1 cells and reinforce Th2 response in consequence [120]. However, Korn et al. have revealed that Th1 and Th2 cells release a relatively small amount of IL-21 compared to developing Th17 cells, which are the main source of this cytokine [121]. IL-21 promotes Th17 differentiation and seems to function analogously to the effects of Th1 and Th2 cytokines on their respective lineages. It is also demonstrated that IL-21 acts as an autocrine regulator of IL-17A and IL-17F production in a STAT3-dependent manner [122]. IL-21 enhances B cell differentiation and proliferation, and production of IgG [105]. Research revealed the increased level of IL-6 and IL-21, associated with the tendency to release more IL-17A, only in NMO patients, particularly in AQP4-IgG-seropositive ones, not in patients with MS [105]. For proper Th17 differentiation the combined actions of IL-6 and IL-21 are necessary, which are extremely dependent on IL-23 cytokine [123]. In agreement with this study, Linhares et al. have demonstrated that increased Th17-related cytokine production by activated CD4+ T cells from NMO patients in the remission phase was directly related to enhanced release of IL-6 and IL-23 by LPS-stimulated monocytes [112]. Furthermore, it is demonstrated that the expression of Th1 and Th17-related cytokines are upregulated in NMO, and the dominant Th17-related response in NMO is positively correlated with neurological disability [112]. The correlation between CSF IL-21 levels in NMO patients and complement has been revealed, which confirmed the association of this cytokine with the humoral immune activity in NMO. However, this study did not report significant differences in IL-21 levels between NMO and MS cohorts [124].

Human G-CSF is produced mainly by macrophages and monocytes; however, it is also released by endothelial cells, fibroblasts, and bone marrow stromal cells [125]. The receptor for G-CSF (G-CSFR) is expressed mostly on neutrophils and bone marrow precursor cells, which undergo proliferation and finally differentiate into the mature granulocytes [126]. Th17-derived IL-17A is a powerful inflammatory cytokine that works on a diversity of cell types, including fibroblasts, endothelial cells, and epithelial cells to induce the expression of pro-inflammatory cytokines, such as IL-6, TNF, and G-CSF, all of which strongly co-operate to produce a robust inflammatory response [127,128]. Uzawa et al. conducted an extensive CSF samples analysis in the context of differences in pro-inflammatory cytokines panel among NMO and MS patients. They have demonstrated an increased level of IL-6, IL-8, IL-13, and G-CSF in NMO in comparison to MS, while in MS they revealed enhanced concentrations of IL-9, GM-CSF, macrophage inflammatory protein (MIP)-1β and TNF-α in comparison to NMO patients. Furthermore, the CSF IL-6 level in NMO had a significant correlation with the CSF GFAP level, and a weak positive correlation with AQP4-IgG level. The levels of IL-6 and GFAP were increased in the CSF in patients during the first NMO attack and their sensitivities (76.9% and 84.6%, respectively) were similar to those of AQP4-IgG in the serum. They have proposed that NMO patients have elevated CSF cytokines and chemokines associated with Th17 (IL-6, IL-8, and G-CSF), Th2 (IL-1, IL-10, and IL-13) and B cell axes (CXCL13 and IL-21), which contrasts with MS, which is mainly a Th1-dominant disease [129]. In vivo study demonstrated that administration of G-CSF may enhance NMO lesions in mice [130]. Jacob et al. for the first time reported a patient in whom the first episode of NMO was exacerbated after inadvertent administration of G-CSF. The post-mortem study of CNS white matter reported that neurons in and around NMO lesions revealed markedly increased expression of G-CSF in neurons located in and around the lesions, with little or no expression in MS lesions or normal white matter. This study supports a harmful role of G-CSF only in the case of NMO and it is postulated that high G-CSF expression is not a general feature of CNS damage, but it is tissue-specific for NMO pathogenesis [131].

IL-6 has an important role in regulating the balance between pro-inflammatory Th17 cells and Treg [98]. Furthermore, Rincon et al. have shown that IL-6 can additionally modulate the Th1/Th2 balance towards Th2 [132]. Research results revealed the significance of IL-6 in both NMO and MS pathogenesis and the correlation between this cytokine and clinical and laboratory findings [133,134]. The following studies confirmed the crucial role of this pleiotropic cytokine in NMO pathogenesis. The study conducted by Uzawa et al. has demonstrated that the CSF IL-6 level was significantly higher in NMO patients than in patients with MS (*p* = 0.001) and other neurological diseases (*p* = 0.001). More importantly, enhanced CSF levels of IL-6 in only NMO support the view of different pathophysiology of NMO and MS [135]. It is suggested that IL-6 may be useful to predict the clinical course of NMO and differentiate it from MS, other neurological non-inflammatory diseases, or healthy subjects [65,129,136]. Furthermore, IL-6 can increase the survival of plasmablasts that produce anti-AQP4-IgG, which results in further tissue lesions [95]. The optimal cut-off CSF IL-6 point has been found as 7.8 pg/mL for diagnosing NMO [135]. On the other hand, it has been suggested that IL-6 is a candidate for a rather non-specific prognostic biomarker as elevated levels of this cytokine are also associated with neuroinflammatory diseases other than NMO [137].

Analysis of the correlation between Th17 and Th22 cells in patients with NMO and MS demonstrated that the proportion of Th22 and Th17 was increased in these patients compared to healthy subjects. Moreover, the concentration of Th22 was correlated with Th17 in NMO patients, which may indicate a synergistic action of these cells [138]. Th22 cells were elevated in NMO patients compared with MS, similarly to Th17 [105,139]. Although the involvement of Th22 cells in NMO and MS pathogenesis is largely undiscovered, it has been demonstrated that IL-22 activates signal transducer and STAT3 pathway. It probably results in the activation of Th22 and Th17 cells [138,139]. One study revealed that IL-22, which influences the Th1/Th17 axis [140], was significantly lower (*p* < 0.0001) in NMOSD patients than in the healthy control group [141].

HMGB1 is released by damaged and inflammatory cells (including macrophages, neutrophils, dendritic cells). It can act as a pro-inflammatory cytokine and plays a role in inflammatory-mediated autoimmune disorders [142,143]. The increased expression of HMGB1 and its receptors on resident microglia and activated macrophages might enhance the inflammation process based on a positive feedback loop [144]. HMGB1 may contribute to Th17 cells differentiation and production of IL-17A and is associated with neuroinflammation during the relapse in demyelinating inflammatory disorders [145]. IL-17A/IL-8 axis activation in CSF and enhanced level of HMGB1 in the plasma of optico-spinal multiple sclerosis patients have also been determined [92]. The expression of HGMB1 was elevated in the CSF of NMO patients compared to MS and positively correlated with IL-17A and IL-6. Taking this into consideration as well as the fact that, as mentioned earlier, IL-6 regulates Th17/Treg balance, it is suggested that HMGB1 contributes to inhibition of the Treg activity and the exacerbation of the autoimmune inflammation in NMO patients [144]. The significant role of HMGB1 in NMO inflammation and damage of astrocytes has been confirmed by Uzawa et al.; however, they reported that primarily increased levels of HMGB1 in CSF and serum of NMO patients (vs. MS) decreased after treatment [143].

OPN is an acidic phosphoprotein particularly associated with Th1 and Th17 pathways and differentially expressed in NMO and MS. Many studies have demonstrated that OPN also critically contributes to the development of Th1 pathway-mediated immunity and disease, as well as is essential for the promotion of robust Th1 responses [146]. OPN is reported in activated CD4+ T cells and possibly promotes the pro-inflammatory response of Th1 and Th17. Furthermore, similarly to IL-1β and IL-6, it is demonstrated that OPN induces preferent differentiation of T cells towards Th17 subtype [147]. OPN is also supposed to play a key role in the development of autoimmune diseases due to its ability to stimulate macrophages to generates IL-12, as well as inhibit IL-10 production, which enables differentiation of T cells into Th1 subtype [148]. On the other hand, as a result of the mentioned processes, the level of IL-27 diminishes, which in turn favors the differentiation of T cells into Th17. The influence of OPN on disease exacerbations has been reported in EAE [149]. Dendritic cells expressed OPN and stimulated CD4+ cells to produce IL-17A [148]. The concentrations of plasma OPN in NMO and MS were similar and significantly higher than in healthy donors. In both NMO and MS patients, the levels of OPN were significantly increased in the relapse phase and decreased in remission. They were correlated with EDDS, which suggests its influence on the activity and progression of the disease [150,151]. To investigate potential differences in OPN levels in CSF among NMO and MS, Kariya et al. have measured the levels in patients with anti-AQP4-IgG-positive NMO and with MS (all at the relapse phase), as well as in the control group. They have shown that OPN is significantly enhanced in the CSF of NMO patients in comparison to MS patients. Immunohistochemical analyses demonstrated that OPN was noticeably increased in the cerebral white matter of NMO patients and released by neurons, oligodendrocytes, and astrocytes, as well as infiltrating macrophages. They also reported that the interplay of the CSF OPN in NMO patients with integrin αvβ3 promoted macrophage chemotaxis by activating phosphoinositide 3-kinase and MEK1/2 signaling pathways [152].

## 4. Conclusions

The discovery of anti-AQP4 antibodies initiated the concept of NMO as a separate disease from MS and only confirmed different autoimmune etiology of NMO. The course of this disease is nowadays more and more effectively modified by available therapies; however, it is still crucial to establish the diagnosis early, as treatment strategies in the case of NMO are different to those used in MS. Although the general clinical picture of NMO disease is well established, many atypical cases are reported, leading to significant diagnostic issues. The histopathological changes distinguishing NMO from MS include predominant astrocyte damage, while extensive demyelination and neuronal loss are found as characteristic of MS; however, it is known that demyelinating changes in the brain do not exclude the diagnosis of NMO. Searching for specific discriminatory markers is crucial for developing novel diagnostic tools and therapies in NMO and MS. The histopathological changes distinguishing NMO from MS include predominant astrocyte damage, while extensive demyelination and neuronal loss are found characteristic of MS; however, it is known that demyelinating changes in the brain do not exclude the diagnosis of NMO. Searching for specific differentiating markers is crucial for developing novel diagnostic tools and therapies in NMO and MS. Recent outcomes expand the understanding of NMO and MS inflammation markers; however, further research must clarify their diagnostic accuracy and specificity. It should be noted that these two diseases are generally characterized by neuroinflammation, so the expression of some inflammatory mediators might be non-specific.

It is suggested that Th17 cells and Th17-related mediators play an essential role in the immunopathogenesis of both NMO and MS. Emerging findings indicate that the markers allowing differentiation between NMO, MS, and healthy individuals include factors specific to the Th17 pathway, with IL-6 being particularly important. It appears that detailed studies of Th17-related pathways may yield promising results leading to precise differentiation of MS and NMO and faster and more effective therapy.

## Figures and Tables

**Table 1:** Features differentiating MS from NMO [87,88,89,90].

Features	MS	NMO
Prevalence	39.5 per 100,000	0.5–4.4 per 100,000
Latitude gradient	Scandinavian and Atlantic Coast/Central Europe population	Far Eastern population
Female (%)	60–70%	80–90%
Gender ratio (female:male)	2–3:1	4–9:1
Race/ethnic variation	More common in white populations (Caucasians)	More common in non-white populations (Africans, South Americans, and Asians)
Age at onset (years)	20–40	40–60
Median age of onset (years)	28	39
Co-existent autoimmune disease	Rare	Frequent
Distribution of symptoms	Any white-matter track	Optic nerves and spinal cord
Tissue involvement	White matter	White and gray matter
Type of leukocyte infiltrating to the CNS	T and B lymphocytes	Neutrophils and eosinophils
Course of disease	−85% RRMS −15% PPMS	−80–90% relapsing course, −10–20% monophasic
Progressive course	Very often	Very rare
Attack severity	Often mild	Often severe
Spinal cord MRI	Short-segment peripheral cord lesions, partial cord syndrome, asymptomatic	Longitudinally extensive central necrotic lesions, complete cord syndrome, acute T1 hypointensity, symptomatic
Brain MRI	Usually normal with non-specific changes, presence of Dawson’s finger, juxtacortical lesions	Multiple periventricular white-matter lesions, extensive hemispheric lesions
Optic neuritis	Short anterior segment inflammation, unilateral, good recovery	Long posterior segment inflammation, bilateral, poor recovery
Pleocytosis (presence lymphocytes in CSF)	Present	Rarely
Area postrema syndrome	Almost never	Common
Diencephalic syndrome	Very rare	Very common
Presence of OCBs (%)	85%	15–30%
Permanent disability	Present in a late progressive phase	Attack-related
Biomarkers in serum	No specific antibodies	AQP4 antibody
Treatment	CIS: siponimod, IFN-β-1a, PegIFN-β-1a, IFN-β-1b; RRMS: ocrelizumab, ofatumumab PMS: ocrelizumab; SPMS: mitoxantrone, ozanimod	Azathioprine, rituximab, mycophenolate mofetil, methotrexate, mitoxantrone, and cyclophosphamide
Hormonal disturbances	Very rare	Very common
Typical symptoms	Sensory loss or paresthesia, optic neuritis, limb and facial weakness, visual blurring due to diplopia, ataxia, vertigo, bladder dysfunction, pain, cognitive dysfunction sexual dysfunction, fatigue, and seizure	Symptoms of transverse myelitis and optic neuritis, NMO-IgG antibodies present

Abbreviations: AQP4—aquaporin-4; IFN-β-1a—interferon β-1a; MS—multiple sclerosis; NMO—neuromyelitis optica; PegIFN—pegylated interferon β-1a; PPMS—primary-progressive multiple sclerosis; RRMS—relapsing-remitting multiple sclerosis; SPMS—secondary-progressive multiple sclerosis.

## Abbreviations

ADCC	antibody-dependent cellular cytotoxicity
APCs	antigen-presenting cells
AQP4	aquaporin-4
BBB	blood-brain barrier
CIS	clinically isolated syndrome
CNS	central nervous system
CSF	cerebrospinal fluid
DMTs	disease-modifying therapies
EAE	experimental autoimmune encephalomyelitis
EDSS	Expanded Disability Status Scale
EMS	European Medicines Agency
FDA	Food and Drug Administration
G-CSF	granulocyte colony-stimulating factor
G-CSFR	granulocyte colony-stimulating factor receptor
GFAP	glial fibrillary acidic protein
GM-CSF	granulocyte macrophage colony-stimulating factor
HLA	human leukocyte antigen
HMGB1	high-mobility group box protein 1
IA	immunoadsorption
IL	interleukin
IFN	interferon
INF-β-1a	interferon β-1a
LETM	longitudinally extensive transverse myelitis
MMP-9	matrix metalloproteinase-9
MOG	myelin oligodendrocyte glycoprotein
MPO	myeloperoxidase
MRI	magnetic resonance imaging
MS	multiple sclerosis
NMO	neuromyelitis optica
NMOSDs	neuromyelitis optica spectrum disorders
OCBs	oligoclonal bands
OPN	osteopontin
PE	plasma exchange
PegINF	pegylated interferon β-1a
PPMS	primary-progressive multiple sclerosis
RRMS	relapsing-remitting multiple sclerosis
S1P	sphingosine 1-phosphate
SNP	single nucleotide polymorphism
SPMS	secondary-progressive multiple sclerosis
STAT	factor signal transducer and activator of transcription
TGF-β	transforming growth factor-β
Th	T helper cells
TNF-α	tumor necrosis factor α
WHO	World Health Organization

## References

[1] International Statistical Classification of Diseases and Related Health Problems (ICD) by World Health Organization (WHO). https://icd.who.int/browse10/2016/en#/G37.0.

[2] Oshio K., Binder D., Yang B., Schecter S., Verkman A., Manley G. (2004). Expression of Aquaporin Water Channels in Mouse Spinal Cord. Neuroscience.

[3] Lennon A.V., Wingerchuk D.M., Kryzer T.J., Pittock S.J., Lucchinetti C.F., Fujihara K., Nakashima I., Weinshenker B.G. (2004). A Serum Autoantibody Marker of Neuromyelitis Optica: Distinction from Multiple Sclerosis. Lancet.

[4] Huda S., Whittam D., Bhojak M., Chamberlain J., Noonan C., Jacob A., Kneen R. (2019). Neuromyelitis Optica Spectrum Disorders. Clin. Med..

[5] Cree B. (2008). Neuromyelitis Optica: Diagnosis, Pathogenesis, and Treatment. Curr. Neurol. Neurosci. Rep..

[6] Kim S.-H., Kim W., Li X.F., Jung I.-J., Kim H.J. (2012). Does Interferon Beta Treatment Exacerbate Neuromyelitis Optica Spectrum Disorder?. Mult. Scler. J..

[7] Tanaka M., Tanaka K., Komori M. (2009). Interferon-β_1b_. Treatment in Neuromyelitis Optica. Eur. Neurol..

[8] Ip P.-P., Chung C.-Y., Chang C.-C., Lee Y.-F., Wang H.-M., Lian I.-B., Fann C.S.-J., Yang C.-C., Liao F. (2018). Differentiation of Remitting Neuromyelitis Optica Spectrum Disorders from Multiple Sclerosis by Integrating Parameters from Serum Proteins and Lymphocyte Subsets. J. Neuroimmunol..

[9] Wang K.C., Lee C.-L., Chen S.-Y., Chen J.-C., Yang C.-W., Chen S.-J., Tsai C.-P. (2013). Distinct Serum Cytokine Profiles in Neuromyelitis Optica and Multiple Sclerosis. J. Interf. Cytokine Res..

[10] Shimizu Y., Ota K., Kubo S., Kabasawa C., Kobayashi M., Ohashi T., Uchiyama S. (2011). Association of Th1/Th2-Related Chemokine Receptors in Peripheral T Cells with Disease Activity in Patients with Multiple Sclerosis and Neuromyelitis Optica. Eur. Neurol..

[11] Wingerchuk D.M., Lucchinetti C.F. (2007). Comparative Immunopathogenesis of Acute Disseminated Encephalomyelitis, Neuromyelitis Optica, and Multiple Sclerosis. Curr. Opin. Neurol..

[12] Leung S., Liu X., Fang L., Chen X., Guo T., Zhang J. (2010). The Cytokine Milieu in the Interplay of Pathogenic Th1/Th17 Cells and Regulatory T Cells in Autoimmune Disease. Cell. Mol. Immunol..

[13] Amedei A., Prisco D., D’Elios M.M. (2012). Multiple Sclerosis: The Role of Cytokines in Pathogenesis and in Therapies. Int. J. Mol. Sci..

[14] Laman J.D., Thompson E.J., Kappos L. (1998). Balancing the Th1/Th2 Concept in Multiple Sclerosis. Immunol. Today.

[15] Dos Passos G.R., Sato D.K., Becker J., Fujihara K. (2016). Th17 Cells Pathways in Multiple Sclerosis and Neuromyelitis Optica Spectrum Disorders: Pathophysiological and Therapeutic Implications. Mediat. Inflamm..

[16] Waite J.C., Skokos D. (2011). Th17 Response and Inflammatory Autoimmune Diseases. Int. J. Inflamm..

[17] Hou M.-M., Li Y.-F., He L.-L., Li X.-Q., Zhang Y., Zhang S.-X., Li X.-Y. (2019). Proportions of Th17 Cells and Th17-Related Cytokines in Neuromyelitis Optica Spectrum Disorders Patients: A Meta-Analysis. Int. Immunopharmacol..

[18] Li Y., Wang H., Long Y., Lu Z., Hu X. (2011). Increased Memory Th17 Cells in Patients with Neuromyelitis Optica and Multiple Sclerosis. J. Neuroimmunol..

[19] Cierny D., Lehotsky J., Hanyasova S., Michalik J., Kantorova E., Sivak S., Kurca E., Dobrota D., Jesenska L. (2017). The age at onset in Multiple Sclerosis is associated with patient’s prognosis. Bratisl. Lek. Listy..

[20] Pandit L., Asgari N., Apiwattanakul M., Palace J., Paul F., Leite M.I., Kleiter I., Chitnis T. (2015). Demographic and Clinical Features of Neuromyelitis Optica: A Review. Mult. Scler. J..

[21] Marrie R.A., Gryba C. (2013). The Incidence and Prevalence of Neuromyelitis Optica: A Systematic Review. Int. J. MS Care.

[22] Amezcua L., McCauley J.L. (2020). Race and Ethnicity on MS Presentation and Disease Course. Mult. Scler. J..

[23] Borisow N., Kleiter I., Gahlen A., Fischer K., Wernecke K.-D., Pache F., Ruprecht K., Havla J., Krumbholz M., Kümpfel T. (2016). Influence of Female Sex and Fertile Age on Neuromyelitis Optica Spectrum Disorders. Mult. Scler. J..

[24] Miclea A., Salmen A., Zoehner G., Diem L., Kamm C.P., Chaloulos-Iakovidis P., Miclea M., Briner M., Kilidireas K., Stefanis L. (2018). Age-Dependent Variation of Female Preponderance across Different Phenotypes of Multiple Sclerosis: A Retrospective Cross-Sectional Study. CNS Neurosci. Ther..

[25] Ligouri M., Pugliatti M., Giuliani F., De Robertis F., Zimatore G.B., Livrea P., Trojano M., Cocco E., Marrosu M.G. (2000). Age at Onset in Multiple Sclerosis. Neurol. Sci..

[26] Likhar N., Mothe R.K., Esam H., Kinra G., Shah C., Dang A. (2015). Epidemiology and Current Treatment of Neuromyelitis Optica: A Systematic Review. Value Health.

[27] Walton C., King R., Rechtman L., Kaye W., Leray E., Marrie R.A., Robertson N., La Rocca N., Uitdehaag B., Van Der Mei I. (2020). Rising Prevalence of Multiple Sclerosis Worldwide: Insights from the Atlas of MS, Third Edition. Mult. Scler. J..

[28] Lublin F.D., Reingold S.C. (1996). National Multiple Sclerosis Society (USA) Advisory Committee on Clinical Trials of New Agents in Multiple Sclerosis * Defining the Clinical Course of Multiple Sclerosis: Results of an International Survey. Neurology.

[29] Lublin F.D., Reingold S.C., Cohen J.A., Cutter G.R., Sorensen P.S., Thompson A., Wolinsky J.S., Balcer L.J., Banwell B., Barkhof F. (2014). Defining the Clinical Course of Multiple Sclerosis: The 2013 Revisions. Neurology.

[30] Thompson A., Banwell B.L., Barkhof F., Carroll W.M., Coetzee T., Comi G., Correale J., Fazekas F., Filippi M., Freedman M.S. (2017). Diagnosis of Multiple Sclerosis: 2017 Revisions of the McDonald Criteria. Lancet Neurol..

[31] Rzepiński Ł., Zawadka-Kunikowska M., Maciejek Z., Newton J.L., Zalewski P. (2019). Early Clinical Features, Time to Secondary Progression, and Disability Milestones in Polish Multiple Sclerosis Patients. Med. Kaunas Lith..

[32] Weinshenker B.G., Bass B., Rice G.P.A., Noseworthy J., Carriere W., Baskerville J., Ebers G.C. (1989). The Natural History of Multiple Sclerosis: A Geographically Based Study. I. Clinical Course and Disability. Brain.

[33] Wingerchuk D.M., Pittock S.J., Lucchinetti C.F., Lennon V.A., Weinshenker B.G. (2007). A Secondary Progressive Clinical Course is Uncommon in Neuromyelitis Optica. Neurology.

[34] Wingerchuk D.M., Lennon A.V., Pittock S.J., Lucchinetti C.F., Weinshenker B.G. (2006). Revised Diagnostic Criteria for Neuromyelitis Optica. Neurology.

[35] Jarius S., Ruprecht K., Wildemann B., Kuempfel T., Ringelstein M., Geis C., Kleiter I., Kleinschnitz C., Berthele A., Brettschneider J. (2012). Contrasting Disease Patterns in Seropositive and Seronegative Neuromyelitis Optica: A Multicentre Study of 175 Patients. J. Neuroinflamm..

[36] Wingerchuk D.M., Hogancamp W.F., O’Brien P.C., Weinshenker B.G. (1999). The Clinical Course of Neuromyelitis Optica (Devic’s Syndrome). Neurology.

[37] Zantah M., Coyle T.B., Datta D. (2016). Acute Respiratory Failure due to Neuromyelitis Optica Treated Successfully with Plasmapheresis. Case Rep. Pulmonol..

[38] Wingerchuk D.M., Weinshenker B.G. (2005). Neuromyelitis Optica. Curr. Treat. Options Neurol..

[39] Wingerchuk D.M., Banwell B., Bennett J.L., Cabre P., Carroll W., Chitnis T., de Seze J., Fujihara K., Greenberg B., Jacob A. (2015). International Consensus Diagnostic Criteria for Neuromyelitis Optica Spectrum Disorders. Neurology.

[40] Kim H.J., Paul F., Lana-Peixoto M.A., Tenembaum S., Asgari N., Palace J., Klawiter E.C., Sato D.K., de Seze J., Wuerfel J. (2015). MRI Characteristics of Neuromyelitis Optica Spectrum Disorder: An International Update. Neurology.

[41] Carbajal K.S., Mironova Y., Ulrich-Lewis J.T., Kulkarni D., Grifka-Walk H.M., Huber A.K., Shrager P., Giger R.J., Segal B.M. (2015). Th Cell Diversity in Experimental Autoimmune Encephalomyelitis and Multiple Sclerosis. J. Immunol..

[42] Arellano G., Acuña E., Reyes L.I., Ottum P.A., De Sarno P., Villarroel L., Ciampi E., Uribe-San Martín R., Cárcamo C., Naves R. (2017). Th1 and Th17 Cells and Associated Cytokines Discriminate among Clinically Isolated Syndrome and Multiple Sclerosis Phenotypes. Front. Immunol..

[43] McDonough A., Lee R.V., Weinstein J.R. (2017). Microglial Interferon Signaling and White Matter. Neurochem. Res..

[44] Korn T., Bettelli E., Oukka M., Kuchroo V.K. (2009). IL-17 and Th17 Cells. Annu. Rev. Immunol..

[45] Pelletier M., Maggi L., Micheletti A., Lazzeri E., Tamassia N., Costantini C., Cosmi L., Lunardi C., Annunziato F., Romagnani S. (2010). Evidence for a Cross-Talk between Human Neutrophils and Th17 Cells. Blood.

[46] Tabarkiewicz J., Pogoda K., Karczmarczyk A., Pozarowski P., Giannopoulos K. (2015). The Role of IL-17 and Th17 Lymphocytes in Autoimmune Diseases. Arch. Immunol. Et Ther. Exp..

[47] Rauch I., Müller M., Decker T. (2013). The Regulation of Inflammation by Interferons and their STATs. JAK-STAT.

[48] Ma Z., Qin H., Benveniste E.N. (2001). Transcriptional Suppression of Matrix Metalloproteinase-9 Gene Expression by IFN-γ and IFN-β: Critical Role of STAT-1α. J. Immunol..

[49] Obradović H., Krstic J., Kukolj T., Trivanović D., Đorđević I.O., Mojsilović S., Jauković A., Jovčić G., Bugarski D., Santibañez J.F. (2016). Doxycycline Inhibits IL-17-Stimulated MMP-9 Expression by Downregulating ERK1/2 Activation: Implications in Myogenic Differentiation. Mediat. Inflamm..

[50] Kuwabara T., Ishikawa F., Kondo M., Kakiuchi T. (2017). The Role of IL-17 and Related Cytokines in Inflammatory Autoimmune Diseases. Mediat. Inflamm..

[51] Flannigan K.L., Ngo V.L., Geem D., Harusato A., Hirota S.A., Parkos C.A., Lukacs N.W., Nusrat A., Gaboriau-Routhiau V., Cerf-Bensussan N. (2016). IL-17A-Mediated Neutrophil Recruitment Limits Expansion of Segmented Filamentous Bacteria. Mucosal Immunol..

[52] Wang K., Song F., Fernandez-Escobar A., Luo G., Wang J.-H., Sun Y. (2018). The Properties of Cytokines in Multiple Sclerosis: Pros and Cons. Am. J. Med Sci..

[53] Khaibullin T., Ivanova V., Martynova E., Cherepnev G., Khabirov F., Granatov E., Rizvanov A., Khaiboullina S. (2017). Elevated Levels of Proinflammatory Cytokines in Cerebrospinal Fluid of Multiple Sclerosis Patients. Front. Immunol..

[54] Kunkl M., Frascolla S., Amormino C., Volpe E., Tuosto L. (2020). T Helper Cells: The Modulators of Inflammation in Multiple Sclerosis. Cells.

[55] Kalra S., Lowndes C., Durant L., Strange R.C., Al-Araji A., Hawkins C.P., Curnow S.J. (2020). Th17 Cells Increase in RRMS as well as in SPMS, Whereas Various Other Phenotypes of Th17 Increase in RRMS only. Mult. Scler. J.-Exp. Transl. Clin..

[56] Pierson E., Simmons S.B., Castelli L., Goverman J.M. (2012). Mechanisms Regulating Regional Localization of Inflammation during CNS Autoimmunity. Immunol. Rev..

[57] Pereira W.L.D.C.J., Reiche E.M.V., Kallaur A.P., Kaimen-Maciel D.R. (2015). Epidemiological, Clinical, and Immunological Characteristics of Neuromyelitis Optica: A Review. J. Neurol. Sci..

[58] Jasiak-Zatonska M., Kalinowska-Lyszczarz A., Michalak S., Kozubski W. (2016). The Immunology of Neuromyelitis Optica—Current Knowledge, Clinical Implications, Controversies and Future Perspectives. Int. J. Mol. Sci..

[59] Traub J., Häusser-Kinzel S., Weber M.S. (2020). Differential Effects of MS Therapeutics on B Cells—Implications for Their Use and Failure in AQP4-Positive NMOSD Patients. Int. J. Mol. Sci..

[60] Lucchinetti C.F., Mandler R.N., McGavern D., Bruck W., Gleich G., Ransohoff R.M., Trebst C., Weinshenker B., Wingerchuk D., Parisi J.E. (2002). A Role for Humoral Mechanisms in the Pathogenesis of Devic’s Neuromyelitis Optica. Brain.

[61] Papadopoulos M.C., Verkman A. (2012). Aquaporin 4 and Neuromyelitis Optica. Lancet Neurol..

[62] Ratelade J., Zhang H., Saadoun S., Bennett J.L., Papadopoulos M., Verkman A.S. (2012). Neuromyelitis optica IgG and Natural Killer Cells Produce NMO Lesions in Mice without Myelin Loss. Acta Neuropathol..

[63] Obara K., Waliszewska-Prosół M., Budrewicz S., Szewczyk P., Ejma M. (2018). Severe course of neuromyelitis optica in female patient with chronic C hepatitis. Neurol. Neurochir. Pol..

[64] Hendriks J.J., Teunissen C.E., De Vries H.E., Dijkstra C.D. (2005). Macrophages and Neurodegeneration. Brain Res. Rev..

[65] Chihara N., Aranami T., Sato W., Miyazaki Y., Miyake S., Okamoto T., Ogawa M., Toda T., Yamamura T. (2011). Interleukin 6 Signaling Promotes Anti-Aquaporin 4 Autoantibody Production from Plasmablasts in Neuromyelitis Optica. Proc. Natl. Acad. Sci. USA.

[66] Alonso V.R., Rivera J.D.J.F., Garci Y.R., Granados J., Sanchez T., Mena-Hernandez L., Corona T. (2018). Neuromyelitis Optica (NMO IgG+) and Genetic Susceptibility, Potential Ethnic Influences. Central Nerv. Syst. Agents Med. Chem..

[67] Waubant E., Lucas R., Mowry E., Graves J., Olsson T., Alfredsson L., Langer-Gould A. (2019). Environmental and Genetic Risk Factors for MS: An Integrated Review. Ann. Clin. Transl. Neurol..

[68] Eskandarieh S., Nedjat S., Abdollapour I., Azimi A.R., Moghadasi A.N., Asgari N., Sahraian M.A. (2018). Environmental Risk Factors in Neuromyelitis Optica Spectrum Disorder: A Case–Control Study. Acta Neurol. Belg..

[69] Sawcer S., Hellenthal G., Pirinen M., Spencer C.C.A., Patsopoulos N.A., Moutsianas L., Dilthey A., Su Z., Freeman C., Hunt S.E. (2011). Genetic Risk and a Primary Role for Cell-Mediated Immune Mechanisms in Multiple Sclerosis. Nature.

[70] Yoshimura S., Isobe N., Matsushita T., Yonekawa T., Masaki K., Sato S., Kawano Y., Kira J.-I. (2012). Distinct Genetic and Infectious Profiles in Japanese Neuromyelitis Optica Patients According to Anti-Aquaporin 4 Antibody Status. J. Neurol. Neurosurg. Psychiatry.

[71] Matsushita T., Masaki K., Isobe N., Sato S., Yamamoto K., Nakamura Y., Watanabe M., Suenaga T., Kira J. (2020). The Japan Multiple Sclerosis Genetic Consortium Genetic Factors for Susceptibility to and Manifestations of Neuromyelitis Optica. Ann. Clin. Transl. Neurol..

[72] Wingerchuk D.M. (2007). Diagnosis and Treatment of Neuromyelitis Optica. Neurologist.

[73] Bevan C., Gelfand J.M. (2015). Therapeutic Management of Severe Relapses in Multiple Sclerosis. Curr. Treat. Options Neurol..

[74] Gaitán M.I., Correale J. (2019). Multiple Sclerosis Misdiagnosis: A Persistent Problem to Solve. Front. Neurol..

[75] Solomon A.J., Naismith R.T., Cross A.H. (2018). Misdiagnosis of Multiple Sclerosis: Impact of the 2017 McDonald Criteria on Clinical Practice. Neurology.

[76] Borisow N., Mori M., Kuwabara S., Scheel M., Paul F. (2018). Diagnosis and Treatment of NMO Spectrum Disorder and MOG-Encephalomyelitis. Front. Neurol..

[77] Papeix C., Vidal J.-S., de Seze J., Pierrot-Deseilligny C., Tourbah A., Stankoff B., Lebrun-Frenay C., Moreau T., Vermersch P., Fontaine B. (2007). Immunosuppressive Therapy is more Effective than Interferon in Neuromyelitis Optica. Mult. Scler. J..

[78] Kleiter I., Hellwig K., Berthele A., Kümpfel T., Linker R.A., Hartung H.-P., Paul F., Aktas O. (2012). Failure of Natalizumab to Prevent Relapses in Neuromyelitis Optica. Arch. Neurol..

[79] Yoshii F., Moriya Y., Ohnuki T., Ryo M., Takahashi W. (2016). Fingolimod-Induced Leukoencephalopathy in a Patient with Neuromyelitis Optica Spectrum Disorder. Mult. Scler. Relat. Disord..

[80] Gelfand J.M., Cotter J., Klingman J., Huang E., Cree B.A. (2014). Massive CNS Monocytic Infiltration at Autopsy in an Alemtuzumab-treated Patient with NMO. Neurol.-Neuroimmunol. Neuroinflamm..

[81] Wang K.-C., Lin K.-H., Lee T.-C., Lee C.-L., Chen S.-Y., Chen S.-J., Chin L.-T., Tsai C.-P. (2014). Poor Responses to Interferon-Beta Treatment in Patients with Neuromyelitis Optica and Multiple Sclerosis with Long Spinal Cord Lesions. PLoS ONE.

[82] Agasing A.M., Wu Q., Khatri B., Borisow N., Ruprecht K., Brandt A.U., Gawde S., Kumar G., Quinn J.L., Ko R.M. (2020). Transcriptomics and Proteomics Reveal a Cooperation between Interferon and T-Helper 17 Cells in Neuromyelitis Optica. Nat. Commun..

[83] Prasad R., Giri S., Nath N., Singh I., Singh A.K. (2006). 5-Aminoimidazole-4-Carboxamide-1-Beta-4-Ribofuranoside Attenuates Experimental Autoimmune Encephalomyelitis via Modulation of Endothelial–Monocyte Interaction. J. Neurosci. Res..

[84] Kessler R.A., Mealy M.A., Levy M.Z. (2015). Treatment of Neuromyelitis Optica Spectrum Disorder: Acute, Preventive, and Symptomatic. Curr. Treat. Options Neurol..

[85] Goldsmith D., Carrey E.A., Edbury S., Smolenski R., Jagodzinski P., Simmonds H.A. (2004). Mycophenolate Mofetil, an Inhibitor of Inosine Monophosphate Dehydrogenase, Causes a Paradoxical Elevation of GTP in Erythrocytes of Renal Transplant Patients. Clin. Sci..

[86] Collongues N., de Seze J. (2016). An update on the Evidence for the Efficacy and Safety of Rituximab in the Management of Neuromyelitis Optica. Ther. Adv. Neurol. Disord..

[87] Waliszewska-Prosół M., Chojdak-Łukasiewicz J., Budrewicz S., Pokryszko-Dragan A. (2021). Neuromyelitis Optica Spectrum Disorder Treatment—Current and Future Prospects. Int. J. Mol. Sci..

[88] Srikajon J., Siritho S., Ngamsombat C., Prayoonwiwat N., Chirapapaisan N. (2018). Differences in Clinical Features between Optic Neuritis in Neuromyelitis Optica Spectrum Disorders and in Multiple Sclerosis. Mult. Scler. J.-Exp. Transl. Clin..

[89] Lalan S., Khan M., Schlakman B., Penman A., Gatlin J., Herndon R. (2012). Differentiation of Neuromyelitis Optica from Multiple Sclerosis on Spinal Magnetic Resonance Imaging. Int. J. MS Care.

[90] Zhang L., Tian J.-Y., Li B. (2019). Current Immunotherapies for Multiple Sclerosis and Neuromyelitis Optica Spectrum Disorders: The Similarities and Differences. Neuroimmunol. Neuroinflamm..

[91] Komiyama Y., Nakae S., Matsuki T., Nambu A., Ishigame H., Kakuta S., Sudo K., Iwakura Y. (2006). IL-17 Plays an Important Role in the Development of Experimental Autoimmune Encephalomyelitis. J. Immunol..

[92] Ishizu T., Osoegawa M., Mei F.-J., Kikuchi H., Tanaka M., Takakura Y., Minohara M., Murai H., Mihara F., Taniwaki T. (2005). Intrathecal Activation of the IL-17/IL-8 Axis in Opticospinal Multiple Sclerosis. Brain.

[93] Numasaki M., Takahashi H., Tomioka Y., Sasaki H. (2004). Regulatory Roles of IL-17 and IL-17F in G-CSF Production by Lung Microvascular Endothelial Cells Stimulated with IL-1β and/or TNF-α. Immunol. Lett..

[94] Hirai Y., Iyoda M., Shibata T., Kuno Y., Kawaguchi M., Hizawa N., Matsumoto K., Wada Y., Kokubu F., Akizawa T. (2012). IL-17A Stimulates Granulocyte Colony-Stimulating Factor Production via ERK1/2 but not p38 or JNK in Human Renal Proximal Tubular Epithelial Cells. Am. J. Physiol. Physiol..

[95] Fujihara K., Bennett J.L., De Seze J., Haramura M., Kleiter I., Weinshenker B.G., Kang D., Mughal T., Yamamura T. (2020). Interleukin-6 in Neuromyelitis Optica Spectrum Disorder Pathophysiology. Neurol.-Neuroimmunol. Neuroinflamm..

[96] Erta M., Quintana A., Hidalgo J. (2012). Interleukin-6, a Major Cytokine in the Central Nervous System. Int. J. Biol. Sci..

[97] Uzawa A., Masahiro M., Kuwabara S. (2013). Cytokines and Chemokines in Neuromyelitis Optica: Pathogenetic and Therapeutic Implications. Brain Pathol..

[98] Kimura A., Kishimoto T. (2010). IL-6: Regulator of Treg/Th17 balance. Eur. J. Immunol..

[99] Mosmann T.R., Coffman R.L. (1989). TH1 and TH2 Cells: Different Patterns of Lymphokine Secretion Lead to Different Functional Properties. Annu. Rev. Immunol..

[100] Kang H., Li H., Ai N., Liu H., Xu Q., Tao Y., Wei S. (2020). Markedly Elevated Serum Level of T-Helper Cell 17-Related Cytokines/Chemokines in Acute Myelin Oligodendrocyte Glycoprotein Antibody-Associated Optic Neuritis. Front. Neurol..

[101] Cong H., Jiang H., Peng J., Cui S., Liu L., Wang J., Zhang X. (2016). Change of Th17 Lymphocytes and Treg/Th17 in Typical and Atypical Optic Neuritis. PLoS ONE.

[102] Annunziato F., Cosmi L., Santarlasci V., Maggi L., Liotta F., Mazzinghi B., Parente E., Filì L., Ferri S., Frosali F. (2007). Phenotypic and Functional Features of Human Th17 Cells. J. Exp. Med..

[103] Hirota K., Duarte J.H., Veldhoen M., Hornsby E., Li Y., Cua D.J., Ahlfors H., Wilhelm C., Tolaini M., Menzel U. (2011). Fate Mapping of IL-17-Producing T Cells in Inflammatory Responses. Nat. Immunol..

[104] Babaloo Z., Aliparasti M.R., Babaiea F., Almasi S., Baradaran B., Farhoudi M. (2015). The role of Th17 Cells in Patients with Relapsing-Remitting Multiple Sclerosis: Interleukin-17A and Interleukin-17F Serum Levels. Immunol. Lett..

[105] Wang H., Dai Y., Qiu W., Lu Z., Peng F., Wang Y., Bao J., Li Y., Hu X. (2011). Interleukin-17-Secreting T Cells in Neuromyelitis Optica and Multiple Sclerosis during Relapse. J. Clin. Neurosci..

[106] Miossec P. (2009). IL-17 and Th17 Cells in Human Inflammatory Diseases. Microbes Infect..

[107] Milovanovic J., Arsenijevic A., Stojanovic B., Kanjevac T., Arsenijevic D., Radosavljevic G., Milovanovic M., Arsenijevic N. (2020). Interleukin-17 in Chronic Inflammatory Neurological Diseases. Front. Immunol..

[108] Seif F., Khoshmirsafa M., Aazami H., Mohsenzadegan M., Sedighi G., Bahar M. (2017). The Role of JAK-STAT Signaling Pathway and its Regulators in the Fate of T Helper Cells. Cell Commun. Signal..

[109] Brucklacher-Waldert V., Stuerner K., Kolster M., Wolthausen J., Tolosa E. (2009). Phenotypical and Functional Characterization of T Helper 17 Cells in Multiple Sclerosis. Brain.

[110] Lin J., Li X., Xia J. (2015). Th17 Cells in Neuromyelitis Optica Spectrum Disorder: A Review. Int. J. Neurosci..

[111] Varrin-Doyer M., Bs C.M.S., Schulze-Topphoff U., Nelson P.A., Stroud R.M., Cree B.A.C., Zamvil S.S. (2012). Aquaporin 4-Specific T Cells in Neuromyelitis Optica Exhibit a Th17 Bias and Recognize Clostridium ABC Transporter. Ann. Neurol..

[112] Linhares U.C., Schiavoni P.B., Barros P.O., Kasahara T.D.M., Teixeira B., Ferreira T.B., Alvarenga R., Hygino J., Vieira M.M.M., Bittencourt V. (2012). The Ex Vivo Production of IL-6 and IL-21 by CD4+ T Cells is Directly Associated with Neurological Disability in Neuromyelitis Optica Patients. J. Clin. Immunol..

[113] Du L., Chang H., Xu W., Wei Y., Wang Y., Yin L., Zhang X. (2020). Effect of NMO-IgG on the Interleukin-6 Cascade in Astrocytes via Activation of the JAK/STAT3 Signaling Pathway. Life Sci..

[114] Mitsdoerffer M., Kuchroo V., Korn T. (2013). Immunology of Neuromyelitis Optica: A T Cell-B Cell Collaboration. Ann. N. Y. Acad. Sci..

[115] Melamed E., Levy M., Waters P.J., Sato D.K., Bennett J.L., John G.R., Hooper D.C., Saiz A., Bar-Or A., Kim H.J. (2015). Update on Biomarkers in Neuromyelitis Optica. Neurol.-Neuroimmunol. Neuroinflamm..

[116] Matsushita T., Tateishi T., Isobe N., Yonekawa T., Yamasaki R., Matsuse D., Murai H., Kira J.-I. (2013). Characteristic Cerebrospinal Fluid Cytokine/Chemokine Profiles in Neuromyelitis Optica, Relapsing Remitting or Primary Progressive Multiple Sclerosis. PLoS ONE.

[117] Michael B., Elsone L., Griffiths M., Faragher B., Borrow R., Solomon T., Jacob A. (2013). Post-Acute Serum Eosinophil and Neutrophil-Associated Cytokine/Chemokine Profile can Distinguish between Patients with Neuromyelitis Optica and Multiple Sclerosis; and Identifies Potential Pathophysiological Mechanisms—A Pilot Study. Cytokine.

[118] Shi Y., Chen Z., Zhao Z., Yu Y., Fan H., Xu X., Bu X., Gu J. (2019). IL-21 Induces an Imbalance of Th17/Treg Cells in Moderate-to-Severe Plaque Psoriasis Patients. Front. Immunol..

[119] Wan E., Andraski A.B., Spolski R., Li P., Kazemian M., Oh J., Samsel L., Swanson P.A., McGavern D.B., Sampaio E.P. (2015). Opposing Roles of STAT1 and STAT3 in IL-21 Function in CD4+ T Cells. Proc. Natl. Acad. Sci. USA.

[120] Wurster A.L., Rodgers V.L., Satoskar A.R., Whitters M.J., Young D.A., Collins M., Grusby M.J. (2002). Interleukin 21 Is a T Helper (Th) Cell 2 Cytokine that Specifically Inhibits the Differentiation of Naive Th Cells into Interferon γ–producing Th1 Cells. J. Exp. Med..

[121] Korn T., Bettelli E., Gao W., Awasthi A., Jäger A., Strom T.B., Oukka M., Kuchroo V.K. (2007). IL-21 Initiates an Alternative Pathway to Induce Proinflammatory TH17 Cells. Nat. Cell Biol..

[122] Wei L., Laurence A., Elias K., O’Shea J.J. (2007). IL-21 Is Produced by Th17 Cells and Drives IL-17 Production in a STAT3-Dependent Manner. J. Biol. Chem..

[123] Costa V.S., Mattana T.C.C., Da Silva M.E.R. (2010). Unregulated IL-23/IL-17 Immune Response in Autoimmune Diseases. Diabetes Res. Clin. Pr..

[124] Wu A., Zhong X., Wang H., Xu W., Cheng C., Dai Y., Bao J., Qiu W., Lu Z., Hu X. (2012). Cerebrospinal Fluid IL-21 Levels in Neuromyelitis Optica and Multiple Sclerosis. Can. J. Neurol. Sci..

[125] Roberts A.W. (2005). G-CSF: A Key Regulator of Neutrophil Production, but that’s Not All!. Growth Factors.

[126] Mehta H.M., Malandra M., Corey S.J. (2015). G-CSF and GM-CSF in Neutropenia. J. Immunol..

[127] Fouser L.A., Wright J.F., Dunussi-Joannopoulos K., Collins M. (2008). Th17 Cytokines and their Emerging Roles in Inflammation and Autoimmunity. Immunol. Rev..

[128] Park H., Li Z., Yang O.X., Chang S.H., Nurieva R., Wang Y.-H., Wang Y., Hood L., Zhu Z., Tian Q. (2005). A Distinct Lineage of CD4 T Cells Regulates Tissue Inflammation by Producing Interleukin 17. Nat. Immunol..

[129] Uzawa A., Mori M., Sato Y., Masuda S., Kuwabara S. (2011). CSF Interleukin-6 Level Predicts Recovery from Neuromyelitis Optica Relapse. J. Neurol. Neurosurg. Psychiatry.

[130] Saadoun S., Waters P., Bell B.A., Vincent A., Verkman A.S., Papadopoulos M. (2010). Intra-Cerebral Injection of Neuromyelitis Optica Immunoglobulin G and Human Complement Produces Neuromyelitis Optica Lesions in Mice. Brain.

[131] Jacob A., Saadoun S., Kitley J., Leite M., Palace J., Schon F., Papadopoulos M.C. (2012). Detrimental Role of Granulocyte-Colony Stimulating Factor in Neuromyelitis Optica: Clinical Case and Histological Evidence. Mult. Scler. J..

[132] Rincón M., Anguita J., Nakamura T., Fikrig E., Flavell R.A. (1997). Interleukin (IL)-6 Directs the Differentiation of IL-4–Producing CD4+ T Cells. J. Exp. Med..

[133] Uzawa A., Mori M., Arai K., Sato Y., Hayakawa S., Masuda S., Taniguchi J., Kuwabara S. (2010). Cytokine and Chemokine Profiles in Neuromyelitis Optica: Significance of Interleukin-6. Mult. Scler. J..

[134] Uzawa A., Mori M., Ito M., Uchida T., Hayakawa S., Masuda S., Kuwabara S. (2009). Markedly Increased CSF Interleukin-6 Levels in Neuromyelitis Optica, but not in Multiple Sclerosis. J. Neurol..

[135] Uzawa A., Mori M., Masuda H., Ohtani R., Uchida T., Sawai S., Kuwabara S. (2017). Interleukin-6 Analysis of 572 Consecutive CSF Samples from Neurological Disorders: A Special Focus on Neuromyelitis Optica. Clin. Chim. Acta.

[136] Barros P.O., Cassano T., Hygino J., Ferreira T.B., Centurião N., Kasahara T.M., Andrade R.M., Linhares U.C., Andrade A.F.B., Vasconcelos C.C.F. (2015). Prediction of Disease Severity in Neuromyelitis Optica by the Levels of Interleukin (IL)-6 Produced during Remission Phase. Clin. Exp. Immunol..

[137] Bassi M.A.U.S., Iezzi E., Drulovic J., Pekmezovic T., Gilio L., Furlan R., Finardi A., Marfia G.A., Sica F., Centonze D. (2020). IL-6 in the Cerebrospinal Fluid Signals Disease Activity in Multiple Sclerosis. Front. Cell. Neurosci..

[138] Xu W., Li R., Dai Y., Wu A., Wang H., Cheng C., Qiu W., Lu Z., Zhong X., Shu Y. (2013). IL-22 Secreting CD4+ T Cells in the Patients with Neuromyelitis Optica and Multiple Sclerosis. J. Neuroimmunol..

[139] Lejeune D., Dumoutier L., Constantinescu S., Kruijer W., Schuringa J.J., Renauld J.-C. (2002). Interleukin-22 (IL-22) Activates the JAK/STAT, ERK, JNK, and p38 MAP Kinase Pathways in a Rat Hepatoma Cell Line. Pathways that are Shared with and Distinct from IL-10. J. Biol. Chem..

[140] Lindahl H., Olsson T. (2021). Interleukin-22 Influences the Th1/Th17 Axis. Front. Immunol..

[141] Yang H., Han L., Zhou Y., Ding J., Cai Y., Hong R., Hao Y., Zhu D., Shen X., Guan Y. (2019). Lower Serum Interleukin-22 and Interleukin-35 Levels are Associated with Disease Status in Neuromyelitis Optica Spectrum Disorders. CNS Neurosci. Ther..

[142] Naglova H., Bucova M. (2012). HMGB1 and its Physiological and Pathological Roles. Bratisl Lek List..

[143] Uzawa A., Mori M., Masuda S., Muto M., Kuwabara S. (2012). CSF High-Mobility Group Box 1 is Associated with Intrathecal Inflammation and Astrocytic Damage in Neuromyelitis Optica. J. Neurol. Neurosurg. Psychiatry.

[144] Wang H., Wang K., Wang C., Xu F., Zhong X., Qiu W., Hu X. (2013). Cerebrospinal Fluid High-Mobility Group Box Protein 1 in Neuromyelitis Optica and Multiple Sclerosis. Neuroimmunomodulation.

[145] Shi Y., Shotorbani S.S., Su Z., Liu Y., Tong J., Zheng D., Chen J.-G., Liu Y., Xu Y., Jiao Z. (2011). Enhanced HMGB1 Expression May Contribute to Th17 Cells Activation in Rheumatoid Arthritis. Clin. Dev. Immunol..

[146] Denhardt D.T., Noda M., O’Regan A.W., Pavlin D., Berman J.S. (2001). Osteopontin as a Means to Cope with Environmental Insults: Regulation of Inflammation, Tissue Remodeling, and Cell Survival. J. Clin. Investig..

[147] Chen G., Zhang X., Li R., Fang L., Niu X., Zheng Y., He D., Xu R., Zhang J.Z. (2010). Role of Osteopontin in Synovial Th17 Differentiation in Rheumatoid Arthritis. Arthritis Rheum..

[148] Scatena M., Liaw L., Giachelli C.M. (2007). Osteopontin. Arter. Thromb. Vasc. Biol..

[149] Murugaiyan G., Mittal A., Weiner H.L. (2008). Increased Osteopontin Expression in Dendritic Cells Amplifies IL-17 Production by CD4+ T Cells in Experimental Autoimmune Encephalomyelitis and in Multiple Sclerosis. J. Immunol..

[150] Shimizu Y., Ota K., Ikeguchi R., Kubo S., Kabasawa C., Uchiyama S. (2013). Plasma Osteopontin Levels are Associated with Disease Activity in the Patients with Multiple Sclerosis and Neuromyelitis Optica. J. Neuroimmunol..

[151] Comabella M., Pericot I., Goertsches R., Nos C., Castillo M., Navarro J.B., Rio J., Montalban X. (2005). Plasma Osteopontin Levels in Multiple Sclerosis. J. Neuroimmunol..

[152] Kariya Y., Kariya Y., Saito T., Nishiyama S., Honda T., Tanaka K., Yoshida M., Fujihara K., Hashimoto Y. (2015). Increased Cerebrospinal Fluid Osteopontin Levels and its Involvement in Macrophage Infiltration in Neuromyelitis Optica. BBA Clin..

